# Acute Local Cooling to the Lower Body during Recovery Does Not Improve Repeated Vertical Jump Performance

**DOI:** 10.3390/ijerph18095026

**Published:** 2021-05-10

**Authors:** Chansol Hurr

**Affiliations:** Integrative Exercise Physiology Laboratory, Department of Physical Education, Jeonbuk National University, 567 Baekje-daero, Jeollabuk-do, Jeonju-si 54896, Korea; chansolh@jbnu.ac.kr; Tel.: +82-063-270-2835; Fax: +82-063-270-2850

**Keywords:** cryotherapy, exercise performance, vertical jump, recovery, local cooling

## Abstract

BACKGROUND: Local cooling, or cryotherapy, has received attention due to its effects on athlete recovery before or after strenuous exercise. This study seeks to verify the effectiveness of 3 min applications of acute local cooling to the lower extremities between sets of a repeated vertical jump exercise. METHODS: Using a randomized crossover design, twelve subjects performed a total of 3 sets of 30 consecutive maximal vertical jumps and were allowed a recovery period of 5 min after each set. In the recovery period, subjects rested with or without a cooling suit worn on their lower legs. Changes in heart rate, blood lactate, and rate of perceived exertion were assessed. RESULTS: Vertical jump performance steadily decreased during 30 consecutive vertical jumps in all 3 sets; however, no differences in jump performance were observed among the groups. Heart rate, blood lactate, and rate of perceived exertion tended to be lower in the cooling recovery group relative to the control group. CONCLUSION: The current study provides evidence that acute local cooling recovery after a vertical jump exercise may not add any performance benefits but may provide a psychological benefit. The effectiveness of acute local cooling in other functional performances should be addressed in further research.

## 1. Introduction

Athletic competition often provides insufficient recovery time for athletes and this situation can result in physical fatigue and a decrease in performance. In turn, it is critical to develop effective recovery strategies in order to prevent a reduction in performance, particularly in instances when only a short recovery period is possible. One such strategy, cryotherapy, has received attention due to its reported positive effects on alleviating pain or delayed onset muscle soreness (DOMS) [1,2,3] and inflammatory responses [4]. Various types of cryotherapy are now being implemented. These include cold water immersion [3,5,6], topical ice application to relevant body regions [7,8,9], and short exposure to cold air [1,10].

Researchers and practitioners have focused on the recovery effects of cryotherapy for exercise-induced muscle damage. For this reason, eccentric [1,5,7,11] and plyometric exercise [12,13,14] have been extensively investigated as these types of exercise not only induce muscle fatigue, but also result in muscle stiffness, swelling, and soreness (i.e., muscle damage) [15,16]. While most studies have focused on the long-term recovery effects (1–48 h) of cryotherapy, few studies have investigated the short-term recovery effects (<1 h) of cryotherapy between bouts of intense exercise [17].

In general, cooling exercising muscles decreases muscular contractile speed [18,19] and force generating ability [20,21], and alters neuromuscular properties [22,23,24]. All of these effects consequently result in a reduction in exercise performance. Previous studies on applied acute cooling for a period of 10–30 min before intense exercise have shown the detrimental effects of cooling applications on various types of exercise such as the shuttle run, cycling, vertical jump, and isokinetic strength tests [17,25,26,27,28]. Interestingly, acute cooling for 10 min or less has been shown to have a positive effect on muscular strength and fatigue reduction [29,30,31], which is potentially due to altered sensory perception and pain reduction [31,32,33]. This suggests that cooling duration is a key factor in the proper application of local cooling. While these studies focused on isometric and isokinetic muscle contraction exercise, short-term local cooling in response to functional performance has yet to been thoroughly documented.

The primary aim of the current study was to verify the effectiveness of acute local cooling applications to the lower extremities for 3 min between sets of a repeated vertical jump exercise. We have recently detailed a high intensity repeated vertical jump model that provides indexes for fatigue development and recovery [34]. In the current study, we used a customized cooling suit for the lower extremities that was maintained at 7 °C so that subjects were able to tolerate pain sensations from the suit during the 3 min recovery period. We hypothesized that a 3 min cooling session for the lower body would enhance recovery for repeated vertical jump exercise performance.

## 2. Materials and Methods

### 2.1. Ethical Approval

The Institutional Review Board (IRB) at Jeonbuk National University approved all study procedures used in the current experiment (IRB #: JBNU 2019-07-014-003). Participants were given a verbal description of all procedures and informed of the purpose and risks involved in the study. Written consent was obtained before the study began. The study conformed to the provisions of the Declaration of Helsinki.

### 2.2. Subjects and Experimental Protocol

Twelve young, healthy, and recreationally active males (25.4 ± 1.4 years, 178.2 ± 1.7 cm, 79.2 ± 3.2 kg) participated in the current study. Participants were instructed to maintain their regular diet during the participation period. Additionally, they were asked to refrain from strenuous exercise, alcohol, and caffeine consumption during the 24 h preceding the visit. During the experiment, air cushioning shoes were not allowed due to their effects on jump performance.

**Figure 1:**
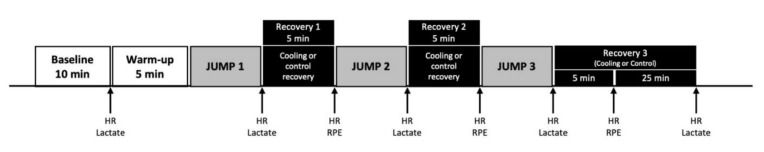
Schematic representation of the experimental protocol. JUMPs 1–3, sets of the vertical jump exercise; HR, heart rate; Lactate, blood lactate; RPE, rate of perceived exertion. During Recoveries 1 and 2, subjects rested with or without the cooling suit in a randomized order. Due to time spent for putting the cooling suit on the test subjects, actual cooling time in Recoveries 1 and 2 was 3 min. Recovery 3 lasted 30 min with or without the cooling suit.

A schematic of the experimental protocol is presented in Figure 1. Subjects visited the laboratory a total of 3 times, consisting of one familiarization visit and two test visits. The familiarization visit was to minimize potential learning effects. During the first visit, participants were informed of the overall experimental protocol, potential risks, and purpose of the study. After completion of a written consent form, participants were familiarized with our repeated vertical jump exercise model and the lower-body cooling suit. Body mass (kg) and height (cm) were also measured during this session.

The following two visits were completed according to a randomized order (i.e., randomized crossover design) with at least a 72 h separation, such that 6 participants performed the control recovery trial in the first visit and vice versa. Any signs of DOMS during the second visit were not reported by participants. Participants had their meals at least 3 h before the visit to prevent stomach upset in response to the exhaustive exercise. Food and water were not given during the entire procedure.

Baseline heart rate (Polar, USA) was measured and averaged during the last 60 s of a 10 min baseline period, and blood lactate (AccuTrend Plus, Roche, Greenwood Village, CO, USA) was measured after a 10 min resting baseline period. Light warm-up exercise was performed using a cycle ergometer (Monark 894E, Vansbro, Sweden) at a speed of 100 RPM for 5 min without resistance to prevent injury during repeated vertical jump. During warm-up exercise, subjects were monitored by experiment assistants, and verbal encouragement was given when needed to maintain 100 RPM. Subjects then performed a total of 3 sets of 30 consecutive maximal countermovement vertical jumps without arm swinging and 5 min recovery periods were given following each set (Figure 1). Previously, we confirmed a linear decline in jump height in the same repeated vertical jump protocol. This assessment represents a valid and reproducible method for muscular fatigue in the lower body [34]. After each set of the vertical jump exercise, subjects rested in a chair for 5 min with or without a cooling suit equipped to the lower legs for either cooling suit recovery or control recovery, respectively. Following the completion of each 5 min recovery period, participants were asked to verbally provide a rate of perceived exertion (RPE) referring to the exercise intensity on a 10-point Borg scale [35]. Following the last set of the vertical jump exercise (JUMP 3), subjects rested for 30 min with or without the cooling suit. For the recovery sessions where the cooling suit was used, two assistants put on and took off the cooling suit on the lower legs of each participant, which took 2 min (i.e., 3 min cooling during 5 min recovery period). A 3 min cooling period was chosen. The reason for this was that a previous literature review revealed that a cooling duration of less than 3 min shows a positive effect size compared to cooling duration longer than 3 min [17]. All other procedures were identical between the two different visits. Time points for heart rate, blood lactate, and RPE measurements are shown in Figure 1. Heart rate was measured during the last 60 s of each stage and averaged for data analysis.

#### 2.2.1. Vertical Jump

Jump duration was measured as an index of jump height by digital vertical jump equipment (TKK-5414, TAKEI, Niigata, Japan). During each set, subjects jumped in synchronization with a metronome with a cadence of 12 beats/min, while the total exercise time of each set was 150 s (i.e., 30 jumps over 150 s). To minimize the effects of arm swing during jumping sessions, both arms were crossed and hands were placed on the shoulders. To minimize the possibility of a feedback effect, subjects were not allowed to see their own jump height measurements during their jumping session.

#### 2.2.2. Cooling Suit

The cooling suit consisted of three parts, respectively, for the thigh, knee, and lower leg (Ice Tube, COOLMEDICS, Korea). The inner surface of the suit was attached to gel-filled silicone cells with Velcro. The gel was a mixture of purified water and nontoxic chemicals. The suit temperature was maintained at 7 °C for at least 1 h when frozen [36]. The silicone cells were prepared in ice water for 1 h and attached to each piece of the cooling suit before use. The cells make contact with the skin and lower the muscle temperature for the areas associated with vertical jump exercise: the quadriceps femoris, the hamstrings, and the gastrocnemius.

#### 2.2.3. Statistical Analysis

Data are expressed as the mean ± SE. Multiple group comparisons were performed using two-way repeated measures analysis of variance (ANOVA) ([repetition or set] × [recovery] or [time] × [recovery condition]), which was followed by Bonferroni post hoc analysis. Significance was set at *p* < 0.05 (Prism 8.3, GraphPad, San Diego, CA, USA).

## 3. Results

### 3.1. Vertical Jump Performance

**Figure 2:**
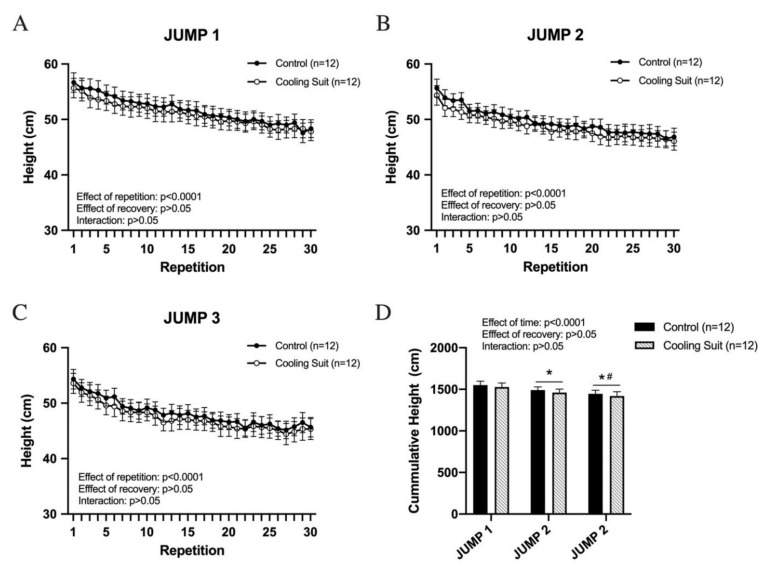
Vertical jump performance. Each set (**A**–**C**) consists of 30 consecutive vertical jumps. Cumulative jump height in each set is presented in (**D**). No significant differences were observed between the control group and the cooling suit group. * *p* < 0.05 vs. JUMP 1, # *p* < 0.05 vs. JUMP 2. Data are presented as the mean ± SE.

In JUMP 1, vertical jump performance steadily decreased from the beginning to the end of the session in both recovery groups. As we expected, no differences were observed (main effect of repetition, *p* < 0.0001; main effect of recovery, *p* > 0.05, interaction (repetition x recovery condition), *p* > 0.05) (Figure 2A). After 5 min of recovery with the suit, however, local cooling did not seem to have an effect on jump performance (main effect of repetition, *p* < 0.0001; main effect of recovery, *p* > 0.05, interaction (repetition × recovery condition), *p* > 0.05) (Figure 2B). In JUMP 3, there was no difference in jump performance between the recovery groups (main effect of repetition, *p* < 0.0001; main effect of recovery, *p* > 0.05, interaction (repetition x recovery condition), *p* > 0.05) (Figure 2C). We also analyzed the cumulative heights for each set (Figure 2D). Jump performance decreased from JUMP 1 to JUMP 3. Cooling suit recovery did not show any positive effects on total exercise (JUMP 1—1539.2 ± 46.9 vs. JUMP 2—1475.5 ± 39.1 vs. JUMP 3—1432.5 ± 46.9 cm, *p* < 0.05 for both conditions combined) (main effect of time, *p* < 0.0001; main effect of recovery condition, *p* > 0.05; interaction (time x recovery condition), *p* > 0.05).

### 3.2. Heart Rate

**Figure 3:**
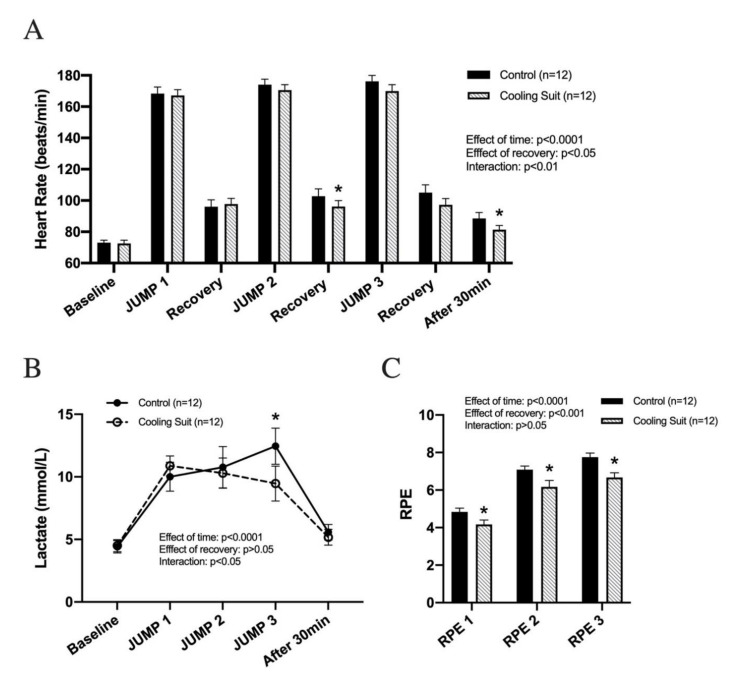
Changes in heart rate (**A**), lactate (**B**), and RPE (**C**). Each set (**A**–**C**) consists of 30 consecutive vertical jumps. * *p* < 0.05 vs. control. Data are presented as the mean ± SE.

As expected, heart rate (HR) dramatically increased after JUMP 1 and partially decreased after the 5 min recovery for both recovery groups (baseline—72.8 ± 1.8 vs. JUMP 1—167.8 ± 3.9 vs. recovery—1 96.9 ± 4.0 bpm; *p* < 0.01 for both conditions combined). No differences were observed between the groups in each recovery session (*p* > 0.05) (Figure 3A). Similar patterns in HR were seen in JUMP 2/Recovery 2 and JUMP 3/Recovery 3 (Recovery—1 96.9 ± 4.0 vs. JUMP 2—172.3 ± 3.5 vs. recovery—2 99.5 ± 4.3 vs. JUMP 3—172.9 ± 3.9 vs. Recovery 3—101.2 ± 4.2 bpm; *p* < 0.01 for both conditions combined). HR during Recoveries 2 and 3, however, tended to be lower for the group using the cooling suit (Figure 3A, control vs. cooling suit recovery, *p* < 0.05 and *p* = 0.06 for Recovery 2 and Recovery 3, respectively). A lower HR was also observed for the cooling suit group relative to the control group in the 30 min period following JUMP 3 (control 88.5 ± 3.9 vs. cooling suit 81.3 ± 2.8 bpm, *p* < 0.05) (main effect of time, *p* < 0.0001; main effect of recovery condition, *p* < 0.05; interaction (time x recovery condition), *p* < 0.01) (Figure 3A).

### 3.3. Blood Lactate

In both recovery groups, blood lactate increased by similar levels after JUMP 1 and JUMP 2 (Figure 3B); however, lactate levels were significantly lower in the cooling suit group when compared to the control group (control—12.5 ± 1.5 vs. cooling suit—9.5 ± 1.4 mmol/L, *p* < 0.05). In the 30 min after JUMP 3, lactate levels returned to their baseline level (baseline—4.5 ± 0.5 vs. after 30 min—5.4 ± 0.6, *p* > 0.05) with no difference observed between the recovery groups (control—5.6 ± 0.6 vs. cooling suit—5.2 ± 0.6, *p* > 0.05) (main effect of time, *p* < 0.0001; main effect of recovery condition, *p* > 0.05; interaction (time × recovery condition), *p* < 0.05) (Figure 3B).

### 3.4. RPE

In both recovery groups, PRE increased from JUMPs 1 to 3 (PRE 1—4.5 ± 0.2 vs. 6.6 ± 0.2 vs. 7.2 ± 0.2, *p* < 0.05 for both conditions combined). As expected, PRE was significantly lower in the cooling suit group in all stages (RPE 1, control—4.8 ± 0.2 vs. cooling suit—4.2 ± 0.2, RPE 2, control—7.1 ± 0.2 vs. cooling suit—6.2 ± 0.3, RPE 3—7.8 ± 0.2 vs. cooling suit—6.7 ± 0.3, *p* < 0.05 for all comparisons) (main effect of time, *p* < 0.0001; main effect of recovery condition, *p* < 0.001; interaction (time x recovery condition), *p* > 0.05) (Figure 3C).

## 4. Discussion

In the present investigation, we evaluated whether the application of acute local cooling between sets of the repeated vertical jump exercise would have a positive effect on recovery and jump performance. Contrary to our hypothesis, the data showed no significant effects on recovery or jump height. Although short-term cooling has been used in sports such as baseball, basketball, and martial arts, the current study provides evidence that acute cooling recovery after a vertical jump exercise may not provide any performance benefits. The effectiveness of acute cooling as a recovery mode in other functional performance should be addressed in further research.

The acute local cooling for exercise performance has been extensively studied for its effectiveness [17]. The general agreement is that local cooling over exercising muscles impairs exercise performance due to (1) muscular contractile speed [18,19], (2) force generating ability [20], and (3) alteration in neuromuscular properties [22]. This is because a slower release of Ca^++^ from sarcoplasmic reticulum decreases muscular contraction and relaxation speed as well as force generating ability in response to decreased muscular temperature [18,22]. Additionally, studies have shown that conduction velocity is reduced in terminal nerve endings. Cooling also decreases conduction velocity in motor neuron terminals and the number of acetylcholine molecules binding to the postsynaptic receptor [20,23,24].

Our own laboratory recently showed that acute cooling recovery for 10 min between bouts of the traditional Wingate anaerobic test (WAnT) reduces neuromuscular activity, and consequently, exercise performance [25]. Similarly, Cross et al. determined that ice immersion of the lower legs for 20 min impairs vertical jump and shuttle run performance in soccer and football athletes [37].

Interestingly, previous studies have shown that acute cooling directly to the skin over the exercising muscle improves exercise performance when applied between sets of intensive exercise [31,32]. Verducci et al. reported that 3 min ice cooling on the arm and shoulder between sets increases 75% 1RM in healthy individuals [31]. The same research team also revealed that 3 min cooling on the arm and shoulder of professional baseball pitchers improves pitching performance [32]. This finding has been practically applied during baseball games, where baseball pitchers put ice pads or suits around their shoulder after every inning. Costello et al. assessed time course changes in muscle and skin temperature in response to 4 min cold water immersion (CWI) and whole-body cryotherapy (WBC) [38]. They reported that muscle temperature 3 cm below the skin begins decreasing 10 min after cooling application while skin temperature drops immediately following the completion of CWI and WBC. This suggests that 3 min cooling is not likely to result in dramatic changes in muscle temperature in the present investigation.

Acute cooling has been found to have positive effects on fatigue and performance such as alteration in sensory perception [28,39] and pain reduction [29,31,32]. Cooling can cause local analgesia and increase pain receptor thresholds [39]. Due to an increased threshold of pain receptors, sensitivity of sensory perception such as perceived pain from exercise-induced fatigue would be lowered. This allows for improvements in exercise performance.

Given the lower RPE in the cooling recovery group (Figure 3C), cooling application in the current study successfully played a role in both sensory perception and pain reduction. Another element of the positive effects of acute cooling is called “The Gate Control Theory”. In this theory, some peripheral stimuli, including cooling and heating, affect different sets of neurons that are in charge of pain transmission [17].

In our study, 3 min cooling recovery did not elicit improvement in repeated vertical jump performance. Given that HR and blood lactate were lower in the cooling recovery group relative to control recovery group (Figure 3A,C), we were able to confirm that our cooling recovery method played a physiological role that was similar to previously reported data [25,40]. In agreement with our data, other studies reported that 3–5 min cooling did not elicit improvement in exercise performance in exercises such as grip strength, single-leg vertical jump, and the shuttle run [26,41]. Considering the muscles involved in jumping, it is plausible that the cooling areas in the current study were insufficient. We applied the cooling suit to quadriceps femoris, hamstrings, and gastrocnemius due to their dominant roles during vertical jump. Based on the countermovement aspect of a vertical jump in our experiment, however, other muscles such as the tibialis anterior and the glutes also play a pivotal role. Additionally, the knee and hip joints are known to be involved in the countermovement of vertical jumps. Thus, since these muscles and joints were not addressed in our cooling recovery process, they could not experience the positive effects of recovery offered by cooling.

## 5. Conclusions

In practice, one should increase muscle temperature before exercise or sports competition by performing warm-ups; muscle cooling before exercise must be avoided. However, acute cooling application has the potential to improve exercise performance as long as it does not decrease muscle temperature. For example, acute cooling would be beneficial in a situation where high intensity repeated exercise is performed in hot environment, which is known to cause an increase in central nervous system-induced fatigue and blood distribution to the skin. In the present study, we failed to show that 3 min acute cooling recovery improves repeated vertical jump performance. In turn, further investigation into the effects of cooling in various environmental conditions or functional performances is needed.

## Data Availability

Not applicable.
